# Development and Concurrent Validity of the Short-Form CogDrisk Dementia Risk Assessment Tool

**DOI:** 10.14283/jpad.2024.108

**Published:** 2024-06-25

**Authors:** Kaarin J. Anstey, M. H. Huque, S. Kootar, R. Eramudugolla, M. Li

**Affiliations:** 1 School of Psychology, University of New South Wales, UNSW, Matthews Building, Kensington, Sydney, NSW 2052 Australia; 2 Neuroscience Research Australia, Randwick, NSW 2031 Australia; 3 UNSW Ageing Futures Institute, University of New South Wales, Sydney, 2052 Australia; 4 Sydney Children’s Hospital, Westmead, NSW 2145 Australia

**Keywords:** Risk assessment, validation, dementia prevention, risk factor

## Abstract

Evidence-based dementia risk assessment is required to inform individual and policy-level dementia risk reduction interventions. We developed the CogDrisk Short Form (CogDrisk-SF) to assess dementia risk factors, for situations where time and resources are limited. To evaluate concurrent validity with the original CogDrisk, we conducted an online survey using a repeated-measures, counterbalanced design. Community dwelling participants (n = 647, 50.1% were female, mean age 62.2 years, age range 40–89) completed the survey. The mean(sd) score for CogDrisk-SF and the CogDrisk was 9.7 (5.3) and 9.9 (5.5), respectively. The intraclass correlation between the risk score obtained from CogDrisk and CogDrisk-SF was 0.92. Fish intake, insomnia and depression had percentage agreements of 79%, 87% and 89% respectively. Other items had >95% agreement except for loneliness (94%), hypertension (94%), cholesterol (93%), atrial fibrillation (91%) and cognitive activity (90%). Very high agreement between the CogDrisk-SF and original CogDrisk shows that CogDrisk-SF is valid for use in research and clinical practice.

**Electronic Supplementary Material:**

Supplementary material is available in the online version of this article at 10.14283/jpad.2024.108.

## Introduction

Given the enormous projected prevalence of dementia globally (1), due to population ageing, there is an urgent need to identify all available methods to reduce risk of dementia. Addressing modifiable risk factors needs to be a key component of prevention strategies, with the Lancet Commission estimating up to 40% of cases may be attributable to twelve risk factors it identified in 2020 (2). In 2019, the World Health Organisation (WHO) also identified nine risk factors for which there is evidence that interventions are beneficial (3). Since the publication of these reports, the literature has continued to grow, as has the publication of statistical risk prediction models, often developed from single cohort studies (4). These statistical models are not necessarily designed with a focus on providing risk information to individuals. There are only a few examples (5–7) of translation of statistical models into practical, evidence-based risk assessment tools for use in clinics and by the general community. The ANU-ADRI was developed in 2013 and is one of the few late-life dementia risk tools that has been widely available through a website and is implemented in brain health clinics (8) and used in population modelling (9). It has a short version (10) which has been used as an outcome in clinical trials (11). However, the evidence base has grown dramatically since the ANU-ADRI (12) was developed.

The CogDrisk (13) was developed and validated (14, 15) to address the need for a low-cost, accessible up to date, late-life dementia risk assessment tool for use by clinicians, the community and policy makers, and to take into account an additional decade of evidence on dementia risk factors (2, 3, 16, 17). In brief, the risk factors included in the CogDrisk were drawn from an umbrella review of 91 meta-analyses reporting pooled estimates for 36 putative risk factors for Alzheimer’s disease, Vascular Dementia, and Any Dementia (16) as well as other recent reports (2, 3). The umbrella review considered midlife and late-life exposure, and the quality of the evidence available for each risk factors and outcome. In developing the CogDrisk (13), new pooled estimates were calculated for diabetes (men and women), mid-life obesity, mid-life cholesterol, stroke (with and without atrial fibrillation) to take into account the most recent evidence. We validated CogDrisk for predicting dementia in four cohort studies from the USA and Sweden (14, 15). The full CogDrisk tool includes 91 items of which 40 are not used directly in the calculation of the risk score. These items are included to support management of health risks in primary care and to provide information to assist in the interpretation of results or recommendations for intervention. For example, the full scale includes additional demographic information that would be used in a clinical assessment, such as questions on ethnicity, First Nations status, languages spoken, and far more dietary information than is included in the algorithm.

### Purpose of the present study

The overall aim of our research program is to translate the highest quality evidence into tools that are of optimal use in the real world for dementia prevention. Given the limited time available for dementia risk assessment in clinical settings, and in large surveys, we identified a need for a minimal dementia risk factor assessment that only includes items contributing to the risk algorithm. We therefore developed a short form of the CogDrisk (CogDrisk-SF) and evaluated its agreement with the full version (concurrent validity). Differences in wording and formatting of responses in questionnaires can affect the response rates to individual items in surveys (18) so this evaluation was necessary to clarify whether the CogDrisk-SF would perform similarly to the CogDrisk in assessment of risk.

## Methods

### Participants and design

Participants were recruited via a survey company Qualtrics, a company that provides survey software and in Australia also provides opt-in market research panels to enable researchers to recruit samples. Qualtrics hold the demographic details of participants and this information is used to recruit samples with specific demographic characteristics. Potential participants (who required internet access) were provided with information on the survey title, expected duration and incentive for completion prior to opening their portal. Incentives are managed by the Qualtrics survey company and are intended as a token of appreciation and are approximately $4 to $5 for a 20-minute survey. We also conducted a pilot study of two independent groups using Qualtrics panels with one group completing the short form (n = 336) and the other completing the long form (n = 369) (participant demographics reported in supplementary material 1). The pilot study findings led to revisions to remove ambiguities in questions on head injury and medical conditions. The revised CogDrisk-SF is found in supplementary material 2.

For this study, a sample of 647 participants was obtained by Qualtrics. The sample was stratified by age (40–64 years, 65 years+), gender (male, female), and order of counterbalancing (SF first vs second). Ethical approval was obtained from the University of New South Wales Human Research Ethics Committee. We used a repeated-measures, counterbalanced design (ratio 1:1) such that each participant received both the CogDrisk and the CogDrisk-SF. These were programmed in the Qualtrics software and the survey was administered online. On consenting to participate in the study, participants received access to the Qualtrics survey that contained either the CogDrisk or the CogDrisk-SF first. Participants completed the CogDrisk-SF before the CogDrisk, or vice versa in a single session, according to a predetermined sequence. This was a within persons design and scores of the CogDrisk-SF were compared to the CogDrisk for the same participants.

### CogDrisk questionnaire

The CogDrisk has been described previously (13). In brief, it includes 91 questions to assess 17 risk factors for dementia and the questions were drawn from previously validated scales. Risk factors include age, gender, years of education, self-reported height and weight (from which BMI is obtained, and WHO categories (19) applied), high cholesterol (>= 6.5 mmol/litre or self-report of being told by a health professional that a person has high cholesterol) (20), diabetes, stroke, traumatic brain injury, hypertension, atrial fibrillation, depression (>20 on the Centre for Epidemiological Studies Depression Scale (21)), physical inactivity (assessed with the International Physical Activity Questionnaire (IPAQ) as < 150 mins per week of moderate to vigorous activity (22)), cognitive engagement, social engagement, diet and smoking. Obesity, hypertension and high cholesterol are only included in the total score for mid-life participants.

### Development of the short form of the CogDrisk

When evaluating the validity of the short form of the ANU-ADRI, accuracy was lost due to shortening of measurement scales such as the IPAQ and social, and cognitive engagement scales (10). We therefore decided to retain all items of measurement scales in CogDrisk. The research team developed the draft short form questionnaire by eliminating items in the CogDrisk that do not contribute to the information used in the scoring algorithm, and reformatting or re-wording responses so that matrix tables and multiple-choice options were used where possible (Table 1). We evaluated the validity of the CogDrisk-SF by comparing the total score, with the CogDrisk total score. We also compared the frequencies of endorsement of individual risk factors between the two versions. In the current study the lowest level of education included in the original CogDrisk development (13) (<8 years) was inadvertently omitted from the Education variable in both versions so this did not affect the comparison between them (Supplementary material).

**Table 1:** Comparison between CogDrisk Standard form and Short Form

**CogDrisk Standard V1.3**	**No of items**	**CogDrisk SF**	**No of items**	**Format change**
Personal information	8	Personal information	3	
Height and Weight	2	Height and Weight	2	
Health conditions	12	Health conditions	6	Dropdown
Sleep	7	Sleep	7	
Mood	10	Mood	10	
Physical Activity	6	Physical Activity	6	
Cognitive Activity	14	Cognitive Activity	13	Matrix
Companionship	4	Companionship	1	Matrix
Diet	24	Diet	1	
Alcohol	2			
Smoking	1	Smoking	1	
Pesticides	1	Pesticides	1	
Total	91		51	

### Statistical power and analysis

In order to calculate sample size, we considered two different research questions: (i) whether both the short and long form have a similar ability to predict dementia and (ii) whether risk factor profiles are equally captured in both the short and long form. As the CogDrisk score was developed from research synthesis which utilized data from older adults’ information (>65 years and older), the CogDrisk scores obtained for middle aged adults are suboptimal. Therefore, to estimate the sample size, we utilized the prevalence of risk factors to quantify the risk profile. We assumed that prevalence of depression, diabetes and social isolation in the short form was 40%, 14% and 41%, respectively based on our prior study (10) and the associated figure for the long form was 35%, 8% and 49% (using unpublished data from self-reported conditions from 6171 participants who consented to have their data saved for research from the CogDrisk website). An intraclass correlation (ICC) of > 0.9 is considered acceptable (23) for agreement of short form with longer form questionnaires. Considering 5% level of significance and 80% power, aiming for an ICC of 0.9, we estimated that sample sizes of 627, 153 and 282 respectively, would be required to detect the assumed difference in prevalence of depression, diabetes, and social isolation, between forms. Therefore, we estimated that a minimum sample size of 632 (rounding up due to age-sex stratification and counterbalancing) would be sufficient to capture the difference in prevalence (if any) for depression, diabetes, and social isolation. To obtain this sample, Qualtrics oversampled individuals and collected information from 647 individuals completing both the surveys. Descriptive statistics, Chi-square test statistics with p-value, % agreement, Cohen’s kappa (95% CI) and Intra-cluster correlation (ICC) were used to evaluate the agreement between versions. ICC was calculated based on multilevel linear and logistic mixed effect models for continuous and categorical variables, respectively. Stata statistical software: Release 16, (College Station TX; StataCorp LLC) was used for sample size calculation and statistical analyses.

## Results

### Characteristics of participants

The sample comprised 647 participants (50.1% female, Mean Age 62.2, age range 40–89) who completed the study online. Of the sample, 16.8% completed the school certificate or equivalent, and 26% completed post school qualifications. The CogDrisk questionnaire took 15.3 minutes, and the CogDrisk-SF questionnaire took 9.8 minutes to complete. Descriptive statistics for the sample are shown in Table 2.

**Table 2:** Agreement of the CogDrisk items between short and long form

**CogDrisk item**	**Short form N=647 (%)**	**Long form N=647 (%)**	**Chi-square (χ**^**2**^**)**	**P-value**	**% Agreement**	**Cohen’s Kappa (95% CI)**
Age: mean (sd)	62.2 (11.9)	62.2 (11.9)				
Age Group, years			0.10	1.00	0.99	0.99 (0.98, 1.00)
40–59	251 (38.8)	251 (38.8)				
60–64	70 (10.8)	70 (10.8)				
65–69	121 (18.7)	121 (18.7)				
70–74	113 (17.5)	113 (17.5)				
75–79	58 (9.0)	56 (8.7)				
80–84	20 (3.1)	21 (3.2)				
85–89	14 (2.2)	15 (2.3)				
Gender			1.00	0.61	1.00	1.00 (0.99, 1.00)
Male	323 (49.9)	323 (49.9)				
Female	324 (50.1)	323 (49.9)				
Other identity	0 (0.0)	1 (0.2)				
Education†			0.02	0.90	0.99	0.98 (0.97, 1.00)
8–12 years	467 (72.2)	465 (71.9)				
>13 years	180 (27.8)	182 (28.1)				
Obesity			0.29	0.96	0.97	0.95 (0.94, 0.97)
Underweight	17 (2.6)	16 (2.5)				
Normal	198 (30.6)	206 (31.8)				
Overweight	198 (30.6)	198 (30.6)				
Obese	234 (36.2)	227 (35.1)				
High Cholesterol			2.53	0.11	0.94	0.83 (0.78, 0.89)
No	441 (68.2)	456 (70.5)				
Yes	176 (27.2)	148 (22.9)				
Missing	30 (4.6)	43 (6.6)				
Diabetes			0.02	0.89	0.96	0.96 (0.93, 0.99)
No	529 (81.8)	509 (78.7)				
Yes	118 (18.2)	117 (18.1)				
Missing	0 (0.0)	21 (3.2)				
Stroke			3.65	0.06	0.98	0.76 (0.64, 0.88)
No	623 (96.3)	595 (92.0)				
Yes	24 (3.7)	38 (5.9)				
Missing	0 (0.0)	14 (2.2)				
TBI			0.03	0.95	0.98	0.96 (0.93, 0.99)
No	440 (68.0)	436 (67.4)				
Yes	183 (28.3)	180 (27.8)				
Missing	24 (3.7)	31 (4.8)				
Hypertension			1.31	0.25	0.94	0.87 (0.84, 0.91)
No	382 (59.0)	356 (55.0)				
Yes	265 (41.0)	281 (43.4)				
Missing	0 (0.0)	10 (1.5)				
Insomnia			1.51	0.22	0.87	0.75 (0.66, 0.80)
No	366 (56.6)	345 (53.3)				
Yes	281 (43.4)	302 (46.7)				
Depression			035	0.56	0.89	0.74 (0.69, 0.80)
No	433 (66.9)	423 (65.4)				
Yes	214 (33.1)	224 (34.6)				
Physical activity			0.05	0.82	0.95	0.89 (0.85, 0.92)
Inactive	268 (41.4)	265 (41.0)				
Active	379 (58.6)	382 (59.0)				
Cognitive activity			0.73	0.69	0.90	0.55 (0.45, 0.65)
Low	572 (88.4)	562 (86.9)				
Middle	70 (10.8)	79 (12.2)				
High	5 (0.8)	6 (0.9)				
loneliness			0.01	0.91	0.94	0.86 (0.82, 0.90)
No	224 (34.6)	224 (34.6)				
Yes	423 (65.4)	423 (65.4)				
Fish serve/ week			1.07	0.90	0.79	0.71 (0.67, 0.76)
0	226 (34.9)	227 (35.1)				
0.5	165 (25.5)	161 (24.9)				
1	141 (21.8)	154 (23.8)				
2.5	101 (15.6)	92 (14.2)				
4	14 (2.2)	13 (2.0)				
Smoking			0.08	0.96	0.99	0.97 (0.95, 0.98)
Current smoker	132 (20.4)	136 (21.0)				
Former smoker	194 (30.0)	194 (30.0)				
Non-smoker	321 (49.6)	317 (49.0)				
Atrial Fibrillation			44.1	0.00	0.91	0.54 (0.44, 0.64)
No	607 (93.8)	534 (82.5)				
Yes	40 (6.2)	97 (15.0)				
Missing	0 (0.0)	16 (2.5)				
CogDrisk Score						
mean (sd)	9.7 (5.3)	9.9 (5.5)				
range	−2.6–27.9	−3.1–28.0				

†Note: highest qualification was converted to year of education as: 8–12 for school certificate or equivalent or higher school certificate (or equivalent) or Trade certificate/apprenticeship or Technician’s certificate/advanced certificate or Certificate other than above or Associate diploma. >13 years if Undergraduate diploma or Bachelor’s or Post graduate diploma/certificate or Higher degree.

### Overall agreement between versions

The total scores were 9.7 (SD=5.3) and 9.9 (SD = 5.5) for the CogDrisk-SF and CogDrisk respectively. The ICC was 0.92.

### Agreement between versions on individual risk factors

The number of participants meeting self-reported criteria for each risk factor, using both the CogDrisk SF and the CogDrisk, is shown in Table 2. Agreement between versions was high (over 78%) for each individual risk factor. Fish intake, insomnia and depression had the largest discrepancies with percentage agreements of 79%, 87% and 89% respectively, due to combining multi-item responses followed by dichotomization of these variables in both short and long form. Other items had >95% agreement between SF and LF except for loneliness (94%), hypertension (94%), high cholesterol (93%), atrial fibrillation (91%) and cognitive activity (90%). There was no statistically significant difference in proportions of the sample reporting each risk factor except for atrial fibrillation. In the long form 15% of the sample endorsed this risk factor, but only 6% endorsed it in the short form. We note that 2.5% of the responses of the long version are missing for this item (participant responses do not know are considered missing). Some measures were identical in each version but did not have perfect agreement between the short and long forms of the overall scale, indicating that people vary in their responses even within one testing occasion. For example, both risk scores use the 10-item CES-D, to assess depressive symptoms. Table 3 reported the ICC (95% CI) of various items/scales of the CogDrisk short form and CogDrisk. The ICC (95% CI) of CogDrisk score between CogDrisk short form and CogDrisk is 0.92 (0.91, 0.94). Results for individual risk factors for men and women are reported in Table 4.

**Table 3:** Intra class correlation (ICC) coefficients between scales or items of the CogDrisk short form and CogDrisk

**Item/Scale**	**ICC**	**95% CI**	
		**Lower**	**Upper**
Age	0.998	0.998	0.998
Cholesterol	0.998	0.997	0.999
Diabetes	1		
Stroke	0.998	0.996	0.999
TBI	1		
Depression	0.996	0.994	0.998
Physical Activity	0.999	0.999	1.000
Cognitive activity	0.748	0.712	0.780
Loneliness	0.999	0.998	0.999
Fish consumption	0.835	0.810	0.857
Smoking	0.975	0.971	0.978
CogDrisk scores	0.924	0.912	0.935

**Table 4:** Distribution of chronic disease conditions among males and females in short and long form

	**Male**		**Female**	
**Variable**	**Short form n=323(%)**	**Long Form n=323 (%)**	**Short Form n=324 (%)**	**Long Form n=323 (%)**
Age Group				
40–59	117 (36.2)	117 (36.2)	134 (41.4)	134 (41.5)
60–64	44 (13.6)	44 (13.6)	26 (8.0)	26 (8.0)
65–69	50 (15.5)	50 (15.5)	71 (21.9)	71 (22.0)
70–74	53 (16.4)	53 (16.4)	60 (18.5)	59 (18.3)
75–79	38 (11.8)	36 (11.1)	20 (6.2)	20 (6.2)
80–84	12 (3.7)	13 (4.0)	8 (2.5)	8 (2.5)
85–89	9 (2.8)	10 (3.1)	5 (1.5)	5 (1.5)
Education				
8–12 years	231 (71.5)	228 (70.6)	236 (72.8)	236 (73.1)
>13 years	92 (28.5)	95 (29.4)	88 (27.2)	87 (26.9)
BMI: mean (sd)	29.5 (11.8)	29.0 (10.1)	29.3 (9.8)	29.2 (16.2)
Obesity				
Underweight	3 (0.9)	3 (0.9)	14 (4.3)	13 (4.0)
Normal	95 (29.4)	97 (30.0)	102 (31.5)	108 (33.4)
Overweight	118 (36.5)	120 (37.2)	79 (24.4)	76 (23.5)
Obese	107 (33.1)	103 (31.9)	129 (39.8)	126 (39.0)
High Cholesterol				
No	213 (65.9)	222 (68.7)	228 (70.4)	234 (72.4)
Yes	92 (28.5)	74 (22.9)	84 (25.9)	74 (22.9)
Missing	18 (5.6)	27 (8.4)	12 (3.7)	15 (4.6)
Diabetes				
No	243 (75.2)	234 (72.4)	286 (88.3)	274 (84.8)
Yes	80 (24.8)	78 (24.1)	38 (11.7)	39 (12.1)
Missing	0 (0.0)	11 (3.4)	0 (0.0)	10 (3.1)
Stroke				
No	311 (96.3)	291 (90.1)	312 (96.3)	303 (93.8)
Yes	12 (3.7)	21 (6.5)	12 (3.7)	17 (5.3)
Missing	0 (0.0)	11 (3.4)	0 (0.0)	3 (0.9)
TBI				
No	194 (60.1)	193 (59.8)	246 (75.9)	242 (74.9)
Yes	117 (36.2)	114 (35.3)	66 (20.4)	66 (20.4)
Missing	12 (3.7)	16 (5.0)	12 (3.7)	15 (4.6)
Hypertension				
No	184 (57.0)	167 (51.7)	199 (61.4)	189 (58.5)
Yes	139 (43.0)	149 (46.1)	125 (38.6)	131 (40.6)
Missing	0 (0.0)	7 (2.2)	0 (0.0)	3 (0.9)
Insomnia				
No	193 (59.8)	184 (57.0)	172 (53.1)	160 (49.5)
Yes	130 (40.2)	139 (43.0)	152 (46.9)	163 (50.5)
Depression				
No	227 (70.3)	222 (68.7)	207 (63.9)	202 (62.5)
Yes	96 (29.7)	101 (31.3)	117 (36.1)	121 (37.5)
Physical activity				
Inactive	111 (34.4)	107 (33.1)	158 (48.8)	158 (48.9)
Active	212 (65.6)	216 (66.9)	166 (51.2)	165 (51.1)
Cognitive activity				
Low	286 (88.5)	285 (88.2)	286 (88.3)	277 (85.8)
Middle	35 (10.8)	35 (10.8)	35 (10.8)	44 (13.6)
High	2 (0.6)	3 (0.9)	3 (0.9)	2 (0.6)
loneliness				
No	104 (32.2)	103 (31.9)	119 (36.7)	120 (37.2)
Yes	219 (67.8)	220 (68.1)	205 (63.3)	203 (62.8)
Fish serve/ week				
0	115 (35.6)	118 (36.5)	110 (34.0)	108 (33.4)
0.5	84 (26.0)	76 (23.5)	82 (25.3)	85 (26.3)
1	71 (22.0)	79 (24.5)	70 (21.6)	76 (23.5)
2.5	47 (14.6)	44 (13.6)	55 (17.0)	48 (14.9)
4	6 (1.9)	6 (1.9)	7 (2.2)	6 (1.9)
Smoking				
Current smoker	71 (22.0)	74 (22.9)	61 (18.8)	62 (19.2)
Former smoker	104 (32.2)	103 (31.9)	90 (27.8)	90 (27.9)
Non smoker	148 (45.8)	146 (45.2)	173 (53.4)	171 (52.9)
Atrial Fibrillation				
No	302 (93.5)	263 (81.4)	305 (94.1)	271 (83.9)
Yes	21 (6.5)	47 (14.6)	19 (5.9)	49 (15.2)
Missing	0 (0.0)	13 (4.0)	0 (0.0)	3 (0.9)
CogDrisk Score				
mean (sd)	10.3 (5.8)	10.5 (5.9)	9.1 (4.8)	9.3 (5.0)
range	−2.0 to 27.9	−3.1 to 27.8	−2.6 to 26.0	−2.6 to 28.0

## Discussion

There is now a large amount of evidence on risk factors for dementia (2, 3), which needs to be translated into meaningful tools and interventions that enable individuals and communities to evaluate their modifiable risk factors for dementia (24). CogDrisk was developed to meet this need and the short form addresses the further need for a brief version of the assessment. Our study found very high agreement (25) between the CogDrisk-SF and CogDrisk, showing that CogDrisk-SF is valid for use in research and clinical practice. The only other short form of a dementia risk tool that we have identified is the ANU Alzheimer’s Disease Risk Index Short Form, also developed by our team. That tool was developed specifically to assess measures for Alzheimer’s disease (12) and used a similar methodology to the CogDrisk. Our findings show that at the individual item and scale level there is some variability within participants even within one occasion of measurement. There was variation in responses for identical versions of questionnaires (e.g. the CES-D 10 item depression scale) indicating that there is some response variability which is inevitable. This is informative for risk assessment in general.

The largest difference between versions was seen for assessment of self-reported atrial fibrillation. This is likely due to the differences in wording. In the long form of CogDrisk there is more explanation of atrial fibrillation given in the question i.e. Have you ever been told by your doctor that you have a heart condition like atrial fibrillation/arrhythmias (irregular heartbeats) with/without stroke? (Yes/No/Don’t know). Whereas in the short form it was presented as one of a list of medical conditions that the respondent could select, which captured fewer positive responses. As a result, we propose to add ‘irregular heartbeats’ in brackets after the question on atrial fibrillation in the CogDrisk-SF.

The agreement between individual risk factors in each version was higher than that reported in a study comparing the ANU-Alzheimer’s Disease Risk Index (ANU-ADRI) with a short form of the ANU-ADRI (ANU-ADRI-SF) (10). The agreement between the ANU-ADRI-SF and the ANU-ADRI for fish consumption was ICC=0.772 (0.631–0.849) whereas the ICC for the agreement between the CogDrisk-SF and CogDrisk for fish consumption was 0.835 (0.810–0.857). Most notable, the ICC for cognitive activity was 0.748 (0.712, 0.780) for the CogDrisk forms, in comparison to 0.031 (−0.084 to 0.147) for the ANU-ADRI forms. The higher concurrent validity of the CogDrisk is likely due to retaining the full-length scales for most measures in the CogDrisk-SF. The current study included a larger sample size compared with the ANU-ADRI-SF (10) and other research that compared reliability of the ANU-ADRI in cross-cultural populations (26, 27). The larger ICC obtained in this paper may be due to the large sample size.

The current study had both strengths and limitations. Sourcing participants from a survey company may not reflect the diversity in the population and may introduce sources of bias related to characteristics of people who volunteer for such studies. The within-person design mitigated this to some extent. The experience of completion of the survey via Qualtrics software may differ from administration in a clinical setting or via the CogDrisk website. The questionnaire was presented in English which does not allow for evaluation of the questionnaire instrument in other languages. Strengths included the relatively large sample size and conduct of a pilot study.

We conclude that the CogDrisk-SF is valid for use in clinical and research settings and provides accurate overall dementia risk assessment. Where greater demographic characterisation of respondents is required, or more detail on alcohol consumption, health conditions, cognitive engagement, diet, and sleep habits, the original CogDrisk is recommended.

## Electronic Supplementary Material

Supplementary Material: Development and concurrent validity of the short-form CogDrisk risk tool

Supplementary material, approximately 88.1 KB.

## Notes

*Ethical standards:* Ethical approval was obtained from the University of New South Wales Human Research Ethics Committee.
