# Supplementary material for: Development and Concurrent Validity of the Short-Form CogDrisk Dementia Risk Assessment Tool
Source: J Prev Alzheimers Dis. 2024 Jun 25;11(6):1751–8. doi: 10.14283/jpad.2024.108 (PMC11573794; doi:10.14283/jpad.2024.108)
Supplement: Supplementary file 1 — Supplementary Material: Development and concurrent validity of the short-form CogDrisk risk tool [file 42414_2024_108_MOESM1_ESM.docx]

**Supplementary Material**

**Development and concurrent validity of the short-form CogDrisk risk tool**

Authors: Kaarin J. Anstey, Scherazad Kootar , Ranmalee Eramudugolla, Meiwei Li, Md Hamidul Huque.

Table S1. Pilot study demographics

|  | Male | | Female | |
| --- | --- | --- | --- | --- |
|  | Long form  n=187 (%) | Short form  n=163 (%) | Long form  n=182 (%) | Short form  n=173 (%) |
| Age group |  |  |  |  |
| 40-59 | 91 (48.7) | 67 (41.1) | 83 (45.6) | 76 (43.9) |
| 60-64 | 19 (10.2) | 21 (12.9) | 19 (10.4) | 15 (8.7) |
| 65-69 | 23 (12.3) | 22 (13.5) | 33 (18.1) | 30 (17.3) |
| 70-74 | 21 (11.2) | 23 (14.1) | 21 (11.5) | 24 (13.9) |
| 75-79 | 29 (15.5) | 20 (12.3) | 20 (11) | 21 (12.1) |
| 80-84 | 3 (1.6) | 8 (4.9) | 3 (1.6) | 6 (3.5) |
| 85-89 | 1 (0.5) | 2 (1.2) | 3 (1.6) | 1 (0.6) |
| Education |  |  |  |  |
| <8 Years | 38 (20.3) | 40 (24.5) | 55 (30.2) | 55 (31.8) |
| 8-12 | 52 (27.8) | 61 (37.4) | 68 (37.4) | 61 (35.3) |
| >13 | 97 (51.9) | 62 (38) | 59 (32.4) | 57 (32.9) |
| **Mid-life obesity** |  |  |  |  |
| Underweight | 4 (2.1) | 1 (0.6) | 6 (3.3) | 6 (3.5) |
| Normal | 54 (28.9) | 58 (35.6) | 64 (35.2) | 65 (37.6) |
| Overweight | 90 (48.1) | 50 (30.7) | 47 (25.8) | 46 (26.6) |
| Obese | 37 (19.8) | 51 (31.3) | 62 (34.1) | 50 (28.9) |
| Missing | 2 (1.1) | 3 (1.8) | 3 (1.6) | 6 (3.5) |
| **High Cholesterol** |  |  |  |  |
| Yes | 24 (12.8) | 52 (31.9) | 29 (15.9) | 52 (30.1) |
| Missing | 7 (3.7) | 3 (1.8) | 8 (4.4) | 5 (2.9) |
| **Loneliness** | 138 (73.8) | 113 (69.3) | 111 (61) | 105 (60.7) |
| **Diabetes** |  |  |  |  |
| Yes | 38 (20.3) | 31 (19) | 27 (14.8) | 19 (11) |
| Missing | 5 (2.7) | 0 (0) | 0 (0) | 0 (0) |
| **Stroke** |  |  |  |  |
| Yes | 8 (4.3) | 6 (3.7) | 8 (4.4) | 5 (2.9) |
| Missing | 2 (1.1) | 0 (0) | 4 (2.2) | 0 (0) |
| **Head Injury** |  |  |  |  |
| Yes | 39 (20.9) | 3 (1.8) | 20 (11) | 8 (4.6) |
| Missing | 5 (2.7) | 0 (0) | 5 (2.7) | 0 (0) |
| **High blood pressure** |  |  |  |  |
| Yes | 82 (43.9) | 80 (49.1) | 72 (39.6) | 50 (28.9) |
| Missing | 3 (1.6) | 0 (0) | 1 (0.5) | 0 (0) |
| **Insomnia** | 76 (40.6) | 72 (44.2) | 84 (46.2) | 99 (57.2) |
| **Depression** | 54 (28.9) | 48 (29.4) | 64 (35.2) | 74 (42.8) |
| **Atrial Fibrillation** |  |  |  |  |
| Yes | 21 (11.2) | 10 (6.1) | 17 (9.3) | 6 (3.5) |
| Missing | 4 (2.1) | 0 (0) | 6 (3.3) | 0 (0) |
| **Physical activity** |  |  |  |  |
| Active | 137 (73.3) | 121 (74.2) | 121 (66.5) | 119 (68.8) |
| Inactive | 50 (26.7) | 42 (25.8) | 61 (33.5) | 54 (31.2) |
| **Cognitive activity** |  |  |  |  |
| Low | 157 (84) | 147 (90.2) | 157 (86.3) | 149 (86.1) |
| Middle | 29 (15.5) | 16 (9.8) | 24 (13.2) | 24 (13.9) |
| High | 1 (0.5) | 0 (0) | 1 (0.5) | 0 (0) |

Table S2 Revisions resulting from CogDrisk validation Pilot study.

| CogDrisk SF Pilot | CogDrisk SF Revised | Revision |
| --- | --- | --- |
| Health conditions (Q7) | History of head Injury (Q8) | 1) Separated from matrix  2) not restricted to medically diagnosed TBI |
| Health conditions (Q7) | Stroke | Include TIA |
|  | Hearing Problems (Q10) | Added question |
| Cognitive Activity (Q31) | Cognitive Activity (Q35) | Included “work time” activity |
