# Supplementary material for: Development and Concurrent Validity of the Short-Form CogDrisk Dementia Risk Assessment Tool
Source: J Prev Alzheimers Dis. 2024 Jun 25;11(6):1751–8. doi: 10.14283/jpad.2024.108 (PMC11573794; doi:10.14283/jpad.2024.108)
Supplement: Supplementary file 2 — Supplementary material, approximately 88.1 KB. [file 42414_2024_108_MOESM2_ESM.docx]

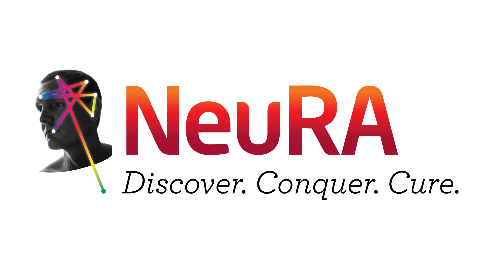


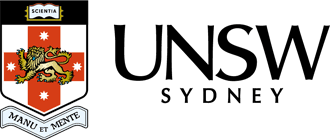


|  | CogDrisk Short Form: A Risk Assessment Questionnaire on Cognitive Health and Dementia Risk Reduction © |
| --- | --- |
|  |  |

**Terms of Use**

The CogDrisk© is publicly copyrighted for use under the Creative Commons license (CC BY-SA) providing the material is attributed to the creators. All use and adaptations must be licensed through UNSW and a licensing agreement can be requested via Professor Kaarin Anstey [k.anstey@unsw.edu.au](mailto:a.morfa@unsw.edu.au).

# **Personal Information**

*In this section, we will ask you for some general information about yourself.*

1. * What is your **age**? ________ (years)
2. * What is your **gender**?

- Male
- Female
- Non-Binary
- Other identity
- Prefer not to say

1. * What was the **highest qualification** that you completed?

- Partially completed primary/elementary school (or equivalent)
- Completed primary/elementary school (or equivalent)
- School certificate (Year 10) (or equivalent)
- Higher school certificate (Year 12) (or equivalent)
- Trade certificate/apprenticeship
- Technician’s certificate/advanced certificate
- Certificate other than above
- Associate diploma
- Undergraduate diploma
- Bachelor’s degree
- Post graduate diploma/certificate
- Higher degree

1. * Enter your **height** in either cm or feet/inches

_____cm or _____feet _____inches

1. * Enter your **weight** in kgs or stones/pounds

____kgs or ______stones ______pounds

# **Your Health**

*The next few questions will be related to your health.*

1. * In the past 2 years, have you ever been told by a doctor or other health professional that you have **high cholesterol** levels, or your cholesterol level is higher than 6.5mmol/L (117mg/dL)?

- Yes
- No
- Don’t know

1. * Have you ever been told by a doctor or other health professional that you have one of the following (select all that apply)

- Diabetes
- High blood pressure
- Stroke or TIA (transient ischaemic attack)
- Atrial fibrillation (irregular heartbeat)
- None of the above

1. Have you ever had a **head injury** or blow to the head that caused you to be dazed, confused, disoriented, or be knocked out?

- Yes, I lost consciousness (knocked out) (Please go to Question 9)
- Yes, I was dazed, confused, or disoriented but did not lose consciousness.
- No
- Don’t know

1. For how long were you unconscious because of your head injury?

- Less than 30mins
- Between 30mins to 24 hours
- More than 24 hours

1. Have you been told by a doctor or health professional that you have **hearing problem**?

- Yes, I was prescribed hearing aids/implant and wear them
- Yes, I was prescribed hearing aids but do not wear them
- No, (Please go to Question 11)
- Don’t know (Please go to Question 11)

1. Do you feel that your hearing is adequate for all purposes?

- Yes
- Cannot hear speech in groups.
- Words are missed in conversation.
- Hearing is a serious problem for me

# **Sleep Questions**

*The next group of questions ask about your sleep* *habits and any problems you may have with sleep.*

For each question, please select the option that best describes your answer.

Please rate the **current** (**i.e., last 2 weeks) severity** of your insomnia problem(s).

| Insomnia Problem | None | Mild | Moderate | Severe | Very Severe |
| --- | --- | --- | --- | --- | --- |
| 1. Difficulty falling asleep | 0 | 1 | 2 | 3 | 4 |
| 1. Difficulty staying asleep | 0 | 1 | 2 | 3 | 4 |
| 1. Problems waking up too early | 0 | 1 | 2 | 3 | 4 |

|  | Not at all | A little | Somewhat | Much | Very much |
| --- | --- | --- | --- | --- | --- |
| 1. How **dissatisfied** are you with your CURRENT sleep pattern? | 0 | 1 | 2 | 3 | 4 |
| 1. How **worried**/**distressed** are you with your CURRENT sleep pattern? | 0 | 1 | 2 | 3 | 4 |
| 1. How **noticeable** to others do you think any sleep problems you may have are in terms of impairing the quality of your life? | 0 | 1 | 2 | 3 | 4 |
| 1. To what extent do any sleep problems you may have INTERFERE with your daily functioning (e.g., daytime fatigue, mood, ability to function at work/daily chores, concentration, memory etc.) **currently**? | 0 | 1 | 2 | 3 | 4 |

# **Feelings**

*The next section asks you about your* ***feelings****. For each of the following statements, please say if you felt that way* ***during the past week****.*

Options are:

**0** Rarely or none of the time (less than 1 day)

**1** Some or a little of the time (1-2 days)

**2** Occasionally or a moderate amount of time (3-4 days)

**3** Most or all of the time (5-7 days)

| Q. No. |  | **Less than one day** | **1-2 days** | **3-4 days** | **5-7 days** |
| --- | --- | --- | --- | --- | --- |
|  | I was bothered by things that usually don’t bother me. |  |  |  |  |
|  | I had trouble keeping my mind on what I was doing. |  |  |  |  |
|  | I felt depressed. |  |  |  |  |
|  | I felt that everything I did was an effort. |  |  |  |  |
|  | I felt hopeful about the future. |  |  |  |  |
|  | I felt fearful. |  |  |  |  |
|  | My sleep was restless. |  |  |  |  |
|  | I was happy. |  |  |  |  |
|  | I felt lonely. |  |  |  |  |
|  | I could not “get going” |  |  |  |  |

# **Daily Physical Activities**

These following questions will ask you about the time you spent being physically active in the **last 7 days**. Please answer each question even if you do not consider yourself to be an active person. Please think about the activities you do at work, as a part of your house and yard work, to get from place to place, and in your spare time for recreation, exercise or sport.

Think about all the **vigorous activities** that you did in the **last 7 days**. **Vigorous** physical activities refer to activities that take hard physical effort and make you breathe much harder than normal. Think *only* about those activities that you did for at least 10 minutes at a time.

1. * During the **last 7 days**, on how many days did you do **vigorous** physical activities like heavy lifting, digging, aerobics, or fast bicycling?

_______ **days per week**

0 **None**

If no vigorous activities, skip to Q31.

1. How much time did you usually spend doing **vigorous** physical activities on one of those days?

_______**hours per day**

______ **minutes per day**

- Don’t know/Not sure

Think about all the **moderate** activities that you did in the **last 7 days**. Moderate activities refer to activities that take moderate physical effort and make you breathe somewhat harder than normal. Think only about those physical activities that you did for at least 10 minutes at a time.

1. * During the **last 7 days**, on how many days did you do **moderate** physical activities like carrying light loads, bicycling at a regular pace, or doubles tennis? Do not include walking.

_______**days per week**

0 **None**

If no moderate physical activities, skip to Q33.

1. How much time did you usually spend doing moderate physical activities on one of those days?

________ **hours per day**

_________**minutes per day**

- Don’t know/Not sure

Think about the time you spent **walking** in the **last 7 days**. This includes at work and at home, walking to travel from place to place, and any other walking that you have done solely for recreation, sport, exercise, or leisure.

1. * During the last **7 days**, on how many days did you walk for at least 10 minutes at a time?

_______**days per week**

0 **None**

If no walking, skip to Q35.

1. How much time did you usually spend walking on one of those days?

________ **hours per day**

_________**minutes per day**

- Don’t know/Not sure

# **Activities you perform during your leisure time and work time (if currently employed).**

*The next section will ask you questions about activities during leisure time and work time (if currently employed).*

During the **past year**, how often did you perform activities in your leisure time and work time (if currently employed)? Please include online activities.

| Questions | **Once a year or less** | **Several times a year** | **Several times a month** | **Several times a week** | **Every day or almost everyday** | **Don’t know** |
| --- | --- | --- | --- | --- | --- | --- |
| 1. Read newspapers, including online |  |  |  |  |  |  |
| 1. Read magazines, including online |  |  |  |  |  |  |
| 1. Read books |  |  |  |  |  |  |
| 1. Play games like checkers or other board games |  |  |  |  |  |  |
| 1. Participate in “brain training” activities including online and computer activities such as Sudoku |  |  |  |  |  |  |
| 1. Write letters/emails |  |  |  |  |  |  |
| 1. Using online social network activities like Facebook/Twitter |  |  |  |  |  |  |
| 1. Engage in intellectually stimulating activities like problem solving, balancing budgets, etc |  |  |  |  |  |  |
| 1. Visit a museum |  |  |  |  |  |  |
| 1. Attend a concert/play/musical |  |  |  |  |  |  |
| 1. Visit a library |  |  |  |  |  |  |

1. * Apart from the above questions, did you participate in other intellectual and cognitively stimulating activities?

***Please comment:***

__________________________________________________________________________________

__________________________________________________________________________________

1. If yes, how often did you participate in the above activities?

| - Every day or almost everyday - Several times a week |
| --- |
| - Several times a month |
| - Several times a year |
| - Once a year or less |
| - Don’t know |

# **Companionship**

*The following questions will ask you about companionship and your feelings.*

1. How often do you feel:

|  | Hardly ever | Some of the time | Often |
| --- | --- | --- | --- |
| a. lack companionship |  |  |  |
| b. left out |  |  |  |
| c. isolated from others |  |  |  |

# **Food and habits**

*In this section, we will ask you questions regarding your diet and habits.*

1. * How often do you eat a serving of fish or seafood that is not deep-fried?

For example, 100g fish fillet or one small can of fish is one serve

- Rarely
- 1-3 times per month
- Once a week
- 2-3 times per week
- 4 or more times per week

1. * Do you, or have you ever, smoked cigarettes, cigars, pipes or any other tobacco products?

- Yes, currently
- Yes, not currently
- Never

# **Environmental exposures**

*The last question is on your exposure to pesticides*

1. * Have you ever been involved with mixing, applying or loading any pesticide, herbicide, weed killers, fumigants or fungicides?

- Yes
- No
- Don’t know
